# Pancreas–Liver–Adipose Axis: Target of Environmental Cadmium Exposure Linked to Metabolic Diseases

**DOI:** 10.3390/toxics11030223

**Published:** 2023-02-26

**Authors:** Diana Moroni-González, Victor Enrique Sarmiento-Ortega, Alfonso Diaz, Eduardo Brambila, Samuel Treviño

**Affiliations:** 1 Laboratory of Chemical-Clinical Investigations, Department of Clinical Chemistry, Faculty of Chemistry Science, Meritorious Autonomous University of Puebla, Ciudad Universitaria, Puebla 72560, Mexico; 2 Department of Pharmacy, Faculty of Chemistry Science, Meritorious Autonomous University of Puebla, 22 South. FCQ9, Ciudad Universitaria, Puebla 72560, Mexico

**Keywords:** cadmium, pancreas, liver, adipose tissue, metabolic diseases

## Abstract

Cadmium has been well recognized as a critical toxic agent in acute and chronic poisoning cases in occupational and nonoccupational settings and environmental exposure situations. Cadmium is released into the environment after natural and anthropogenic activities, particularly in contaminated and industrial areas, causing food pollution. In the body, cadmium has no biological activity, but it accumulates primarily in the liver and kidney, which are considered the main targets of its toxicity, through oxidative stress and inflammation. However, in the last few years, this metal has been linked to metabolic diseases. The pancreas–liver–adipose axis is largely affected by cadmium accumulation. Therefore, this review aims to collect bibliographic information that establishes the basis for understanding the molecular and cellular mechanisms linked to cadmium with carbohydrate, lipids, and endocrine impairments that contribute to developing insulin resistance, metabolic syndrome, prediabetes, and diabetes.

## 1. Introduction

Heavy metals are an environmental threat; every year, there are acute and chronic poisoning cases in occupational and nonoccupational exposure conditions. Epidemiological studies have demonstrated that environmental exposure to low levels of toxic metals develops diverse pathologies. Thus, there is a growing need to understand the molecular events, physiological adaptations, and physiopathological modifications generated by heavy metal exposure. Cadmium (Cd) is a nonessential metal, considered one of the top five most hazardous environmental contaminants by the Agency for Toxic Substances and Disease Registry (ATSDR) [1].

Around 45,000 tons of Cd per year from different sources, such as volcanic emissions, burning fossil fuels, and abrasion of sedimentary rocks, contaminate farmland, water tables, air, and oceans [2]. The United States Geological Survey Mineral Yearbook provides helpful information on global Cd production, where it is explained that the most important sources between 2017–2020 came from Australia, 29%; China, 20%; Germany, 19%; Peru, 11%; and others, 29%. Meanwhile, the refinery production in 2020 and 2021 was led by China, the Republic of Korea, Japan, Canada, Kazakhstan, Russia, and Mexico. Industrial globalization and anthropogenic sources such as phosphate fertilizers (54–58%), atmospheric deposition (39–41%), and sewage sludge (2–5%) contribute to Cd contamination in vegetables, fruits, tubercules, cereals, and legumes [3]. In addition, cigarette smoking may represent an additional Cd source that may equal or exceed that of food.

**Figure 1:**
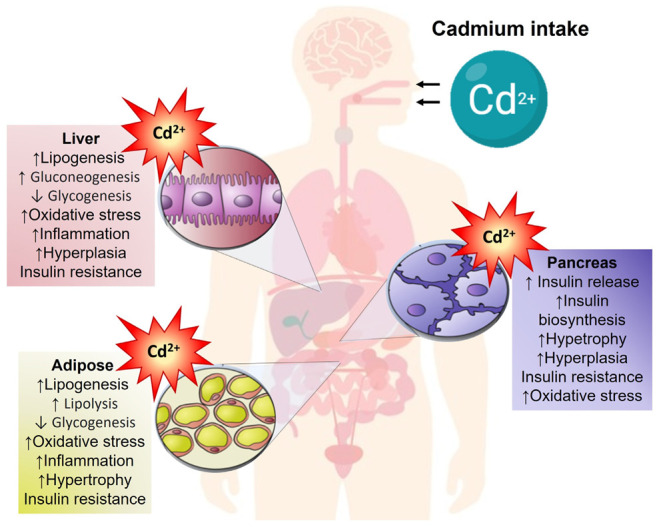
Pancreas–liver–adipose axis in response to cadmium exposure. Cellular response to cadmium toxicity of pancreas, liver, and adipose on metabolism, cell cycle, oxidative stress, and inflammation.

Cadmium poses a significant health risk due to a meager elimination rate (20–40 years); even very low Cd concentrations can accumulate in multiple tissues. The metal cannot degrade to less toxic species and is poorly excreted [4]. The liver, kidney, lungs, testes, prostate, heart, skeletal system, nervous system, and immune system are target organs for Cd toxicity. Furthermore, the principal reservoirs for cadmium are the liver, lungs, bones, and kidneys [5]. Hence, Itai-Itai disease, osteomalacia, osteoporosis, bone fractures, severely impaired renal function, emphysema, anosmia, chronic rhinitis, liver and cardiovascular diseases, testicular dysfunction, and cancer are associated with Cd toxicity. However, adipocyte damage or Langerhans islet dysfunction is rarely mentioned; thus, diabetes and metabolic alterations are not commonly associated with Cd toxicity. The pancreas–liver–adipose axis is central to managing and storing carbohydrates and lipids (Figure 1). However, environmental acute, intermediate, and chronic Cd exposure produces biochemical and hormonal alterations that augment the risk of developing metabolic diseases. This review summarizes studies examining Cd environmental exposure and exposing biological and cellular mechanisms associated with the homeostasis impairment of carbohydrates and lipids focused on related metabolic disorders.

## 2. Minimal Risk Levels of Environmental Cadmium Exposure

According to ATSDR, minimal risk level to humans (MRL) for Cd exposure is defined as an estimate of daily exposure to the metal without an appreciable risk (noncarcinogenic) over a specified duration of exposure. Studies on Cd toxicity in humans and animals via inhalation have established that MRL for acute Cd exposure corresponds to 0.03 µg Cd/m^3^, and chronic duration corresponds to 0.01 µg Cd/m^3^. Meanwhile, acute, intermediate, and chronic oral MRL ranges from 1.12 to 65.6 mg Cd/kg/day, 0.5 µg Cd/kg/day, and 0.1 µg Cd/kg/day, respectively. Adverse effects have been established from dose-response data as the point of departure for MRL. Two MRLs have been identified, the lowest observed adverse effect level (LOAEL) and no observed adverse effect level (NOAEL). Both MRLs are time-depend, concentration (dose), and Cd toxicokinetic [1].

## 3. Cadmium Intake, Absorption, and Distribution

Extensive ranges of Cd concentrations have been reported in foodstuffs from various countries. For example, in Beijing, China, most foodstuffs have Cd concentrations of 0.005–0.100 mg/kg [6]. Estimations of daily Cd intake have been made in several noncontaminated areas. The average daily intake reported for European countries and North America is usually 15–25 μg/day for a 70 kg person [7]. However, the FAO/WHO established a provisional tolerable weekly intake for safe Cd intake. In 1993, Cd intake was set at 400–500 µg per person per week or 140–260 µg/day for over 50 years or 2000 mg of Cd over a lifetime. Presently, tolerable intake is 25 µg per kg body weight per month or 0.83 µg/kg/day or 58 µg/day for a 70 kg person. Exceeding these values breaks the renal threshold, and urinary Cd values increase to above 5.24 µg/g of creatinine [8].

The gastrointestinal tract absorbs 5–10% of the Cd it is exposed to [9]. Meanwhile, the absorption from the respiratory tract is 10–40%. The enterocytes carry Cd through several channels, carriers, and receptors, such as Ca^2+^ channels, transient receptor potential (TRP) channels (e.g., TRPA1, TRPV5/6, TRPML1), solute carriers (e.g., divalent metal transporter 1(DMT1), zinc transporter proteins (ZnT), and zinc-iron permease (ZIP), organic cation transporter 2 (SLC22A2), amino acid/cystine transporter (SLC7A9/SLC3A1), Ferroportin-1 (FPN1/SLC40A1)), and receptors (e.g., Lipocalin-2 Receptor (Lip2-R/SLC22A17) [10]. Then, Cd^2+^ bound to metallothionein (MT) forms the complex Cd-MT and gets into the bloodstream. The Cd-MT complex formation in tissue prevents some toxic effects of metal, but on the other side, the Cd-MT complex can increase cadmium transport to the kidney and liver [11]. Cd may also be bound to low-molecular-weight proteins, such as microglobulins, and only a low proportion to high-molecular-weight proteins, e.g., albumin and transferrin [12]. Consequently, Cd is distributed to different organs and tissues, mainly in the liver (acute exposure) and kidney (chronic exposure).

## 4. Cadmium Toxicity

(a) Interaction with essential biometals. According to the Irving–Williams series, Cd accumulation in organs and tissues can displace zinc, iron, magnesium, manganese, calcium, and selenium from structural domains and active centers of proteins and enzymes. Cd may cause a secondary deficit of oligo-elements and metabolism disruption, which results in functional impairment in many organs. The interaction of Cd with iron and copper is relatively well understood and described because Fenton and Haber–Weiss reactions produce oxidative stress, increasing hydroxyl (•OH) and superoxide (O_2_•^−^) radicals, respectively [13].

(b) Cell signaling impairment. Toxicity could result from Cd interacting with cellular components, such as surface receptors [14]. Several studies have shown cellular signaling pathways impairment after Cd exposure, such as interference with receptors, second messengers, transcription factors, and cell cycle, which cause defects on arrest, differentiation, immortalization, or apoptosis; there have even been reports of post-translational modifications associated with Cd toxicity and cell organelles damage [4]. In addition, Cd can switch on the mitogen-activated protein kinase (MAPK) pathway after inducing phosphorylation on protein kinases for MAPK. Other proteins activated after Cd exposure are transcription factors such as activator protein 1 (AP-1), nuclear factor kappa B (NF-κB), and metal transcription factor -1 (MTF-1), which in turn increase the activity of genes and their expression [4,15].

(c) DNA damage by cadmium. Direct interaction of Cd with DNA causes cancer by epigenetic or indirect genotoxic mechanisms [16]. Cadmium inhibits DNA methylation and impairs gene expression [17]. DNA hypomethylation produces an excessive synthesis of proteins responsible for cell proliferation, which is associated with the development of malignancies. This was found when intracell reactive oxygen species (ROS) increase, which alters the expression of many genes and transcription factors [4,18]. The main overexpression reported in cells exposed to Cd is on c-fos and c-jun, suggesting a possible mechanism responsible for Cd-carcinogenesis.

## 5. Cadmium and Inflammation

It was shown that Cd could modulate an innate immune response [19]. The exact mechanism by which Cd activates an inflammatory response has yet to be fully understood. However, Cd, in a time and concentration-dependent manner, leads to the upregulation of critical pro-inflammatory cytokines and chemokines (TNF-α, IL-1β, IL-8, IL-6, IL-18) and transcription factor genes (Nk-fb1, Nkfb-p65, MYD88) [20]. In vitro studies have demonstrated that nontoxic concentrations of Cd can stimulate IL-6 and IL-8 production through MAPK phosphorylation and NF-kB activation without affecting cell morphology and viability. Likewise, through the MAPK pathway or phosphatidylinositol 3-kinase (PI3K)/Akt signaling, Cd induces cyclooxygenase-2, prostaglandin E2, and macrophage inflammatory protein-2 [21,22]. In addition, it has been reported that Cd activates the ERK1/2 signaling pathway-dependent and NF-κB independent pathways, leading to elevated expression of pro-inflammatory mediators, including IL-8 [23].

## 6. Cadmium and Oxidative Stress

Cadmium cannot generate free radicals directly. Hence, ROS increase after exposure to metal is due to Fenton and Haber–Weiss reactions, as mentioned before. Oxidative stress disturbs the cellular redox balance in favor of the pro-oxidant molecules that disrupt normal cell function [24]. ROS can modulate signal transduction, but the uncontrolled increase carries fatal cell events. Since mitochondria are the major site of ROS production, they are a target of Cd toxicity [25]. ROS production substantially increases in chronic Cd exposure leading to lipid peroxidation, mtDNA cleavage, and impaired ATP generation by mitochondrial damage [26,27]. Cd can unstabilize the semiubiquinones of complex III mitochondrial, which makes them more prone to transfer electrons to molecular oxygen to form superoxide radicals [28,29,30].

Multiprotein complexes of the NOX family are another source of ROS generation. ROS derived from NADPH oxidase plays an essential role in normal cell functioning. However, NADPH oxidase overactivity has been implicated in pathology development and cell injury. ROS overproduction NADPH-dependent reactions after Cd exposure has been found in the liver, kidney, neuronal cells, and multiple cancer cell lines [31,32,33,34]. Since Cd can induce NADPH oxidase overactivity, the production of high free radical levels results in damage or cell death.

## 7. Antioxidative Defense

The cell possesses an antioxidant defense consisting of enzymes, peptides, proteins, and metabolites in each subcellular compartment that minimizes oxidative damage [35]. Cd-induced disturbances in redox balance impair signal transduction cascades, leading to a genic response. The stress-response genes encode MT, heat shock proteins (HSP), glutathione (GSH, the most important cell antioxidant), enzymes (e.g., superoxide dismutase (SOD), catalase (CAT), γ-glutamyl cysteine synthetase, GSH peroxidase (GPx), GSH reductase (GR), GSH transferase (GST)), vitamins (e.g., vitamin C and E), and other antioxidant molecules involved in oxidative stress response [36,37,38]. The most studied transcriptional factors activated by Cd and Cd-induced oxidative stress are metal regulatory transcription factor 1 (MTF1) and NFE2-related factor 2 (NRF2) [39,40,41,42,43,44,45].

Increases and decreases in SOD activity have been described during Cd exposure [46,47]. Discrepancies are attributable to exposure or administration conditions, the organ studied, and specific SOD isoforms localized in cell compartments, Cu/Zn-SOD (cytosolic isoform) and Mn-SOD (mitochondrial isoform) [48,49,50,51]. Cd inhibits Cu/Zn-SOD isoform activity because the Cd-enzyme interaction causes perturbations in the catalytic site [52,53]. After Cd exposure, a decrease in Mn-SOD isoform activity has also been reported [54].

CAT and GPx maintain physiologic cell hydrogen peroxide levels. Whereas CAT activity is in the peroxisomes, cytoplasm, nucleus, and mitochondria [55,56]; the detoxification of H_2_O_2_ by the five isoforms of GPx occurs via GSH oxidation in its active site through selenocysteine [57,58]. According to the literature, CAT activity is better for severe stress situations such as cancer [59,60], while peroxidases act better against low levels of H_2_O_2_, indicating a role in the fine-tuning of signal transduction [61].

Thiol groups of cysteine residues in antioxidative proteins and peptides are vital players in redox sensing and regulation [58,62]. During Cd-induced stress, thiols form Cd–thiol and ROS-thiol complexes that protect, mobilizes, and detoxify cell. Thiols are found in GSH, MT, other small peptides, and proteins that bind Cd in covalent attachments. The GSH system protects cells against xenobiotic compounds and oxidants through GSH as a substrate for GST [63,64,65]. GSH transfers reducing equivalents to GST, and also to GPx, glutaredoxins (Grx), and ascorbate [66]. The GSH levels are maintained by GR activity. Therefore, the GSH, at the same time, neutralizes ROS and eliminates Cd accumulated in the cell, diminishing damage risks [67]. Likewise, MTs that possess numerous thiol groups bind to Cd and other divalent metals and free radicals (such as •OH and O_2_•^−^) [68,69].

## 8. Cadmium and Pancreatic β-Cells

The impaired function of insulin-producing pancreatic β cells in the setting of insulin resistance is the leading underlying cause of type 2 diabetes (T2D). β-cell dysfunction is multifactorial; nevertheless, environmental factors lead to progressive β-cell failure. Studies have shown that acute Cd exposure decreases serum insulin in animals and humans [70,71]. Cadmium is transported into β-cells by DMT, ZIP, and calcium channels. At the same time, zinc exporters (ZnT family widely distributed into β-cell) avoid Cd leak, which explains its accumulation in subcellular compartments, such as insulin granules, endoplasmic reticulum, mitochondria, etc. [72]. In the insulin granules, the hormone is stored as a crystalline hexamer containing two zinc ions and one calcium ion, which prevents amyloidogenesis and degradation [73,74]. Meanwhile, Cd accumulation per se could cause insulin impairment because it may compete with and displace Zn in insulin, promoting hormone degradation. Additionally, amyloid fiber accumulation can be another consequence of Cd toxicity and cell death, thereby diminishing insulin.

Evidence suggests that chronic low-level exposure to Cd in the environment (LOAEL and NOAEL dose) has also been associated with an increased risk of developing dysglycemia and T2D. However, it is unrelated to the death of β-cells [75]. Chronic-Cd exposure in drinking water elicits changes in insulin secretion and insulin resistance development [76,77]. Progressive hyperinsulinemia is an adaptive response by β-cells. However, sustained hyperfunction of β-cells can alter their capacity to downregulate insulin secretion rate over a long-time period [78,79,80]. Most risk variants for T2D in healthy populations act through impairing insulin secretion and, consequently, β-cell dysfunction rather than insulin action that results in insulin resistance. According to this, abnormalities of β-cell function or mass are critical to T2D development [81,82]. The β-cell integrity is essential to an appropriate response to fluctuating metabolic demand for insulin. Glucose is the main regulator of transcription and translation in β-cells, which is necessary for the long-term maintenance of the highly differentiated cell state and the secretory requirements imposed by prolonged elevations of glucose concentration [83,84,85]. The glucose metabolism into β-cells couples the glycolysis and oxidative phosphorylation to increase ATP; in turn, ATP induces K^+^-channel close, which hyperpolarizes β-cells, activating voltage-dependent Ca^2+^ channels that cause mobilization and fusion of insulin granules and, finally, hormone secretion [86]. In each step, Cd accumulation and hyperinsulinemia can interfere. Therefore, β-cells must be prepared to protect against Cd effects. The β-cell adapts in response to fluctuations in demand for insulin. Hyperglycemia increases insulin demands; therefore, β-cells must increase hormone biosynthesis. Insulin synthesis occurs by augmenting transcriptional factors activity, faster insulin translation, and maturation, or via hypertrophy and hyperplasia β-cells, which restore glucose homeostasis [77]. β-cell hypertrophy is associated with p-Ras, p-Raf-1, p-MEK-1, and p-ERK1 pathways [87]. Meanwhile, hyperplasia is linked with c-fos protooncogene, activated by Ras cascade. Ras and c-fos are upregulated by Cd exposure, while the p53 tumor suppression mechanism is inhibited [88,89]. Cadmium may recruit transcription factors usually recruited by zinc, increasing mitogenesis and propagating the replication of damaged DNA. To avoid carcinogenic effects, β-cells increase the pancreatic and duodenal homeobox-1 (PDX-1), a master transcriptional factor induced by the MAPK pathway that plays a critical role in pancreatic development, β-cell differentiation, maintenance of normal function, and regulates the β-cell size and tumorigenesis [90,91]. Moreover, PDX-1 is the principal transcriptional factor in insulin production, and, therefore, it is possible to think that a β-cell adaptation to Cd could be the insulin over-synthesis [91,92].

The regulation and β-cell maintenance concerning mass cell preservation (the integrity of architecture, structure, number, and cell size) and function (physiology) are necessary for T2D not to develop. Serum response factor (SRF), a regulator of myogenic and neurogenic genes, is highly enriched in β cells [93]. SRF is a glucose concentration-sensitive regulator of insulin gene expression [94], and together with the constitutive expression of active peroxisome proliferator-activated receptor (PPARγ), confers glucose homeostasis and the preservation of β-cell mass, preventing apoptosis due to Cd accumulation [95]. The forkhead box class O family (FOXOs) are another transcription factor that participate in β-cell differentiation, proliferation, and survival [96]. FoxO1 is predominantly expressed in the β-cell [97]. FoxO1 accompanies the nuclear exclusion of Pdx-1, suppressing β-cell proliferation [98]. In addition, FoxO1 seems to exert a protective effect against oxidative stress-induced damage, preventing a state of premature β-cell senescence [97,99].

## 9. Protective and Antioxidant β-Cell Capacity

Inflammation and oxidative stress are linked to insulin resistance and Cd accumulation. Exacerbated pro-inflammatory cytokines levels secreted by immune cells infiltrated in Langerhans islet could cause damage or death of β-cell. However, low Cd concentrations in no resolutive conditions, such as chronic exposure at NOAEL or LOAEL dose and sustained hyperglycemia, lead to oxidative stress, causing adaptation and preservation of cell programs [100,101,102]. In vitro, MIN6 cells enlightened Cd toxicity through sub-micromolar concentrations of ceramide that induce IL-6 and IL-1 expression and enhance PGE2 production by COX upregulation [103,104]. Ceramides activate protein kinase C (PKC) and NF-κB pathways, initiating inflammation via NLRP3 inflammasome [105]. Due to β-cells being susceptible to uncontrolled ROS and RNS that generate oxidative distress (nonphysiologic stress), endoplasmic reticulum (ER) stress, DNA damage, and the formation of advanced glycation end products (AGEs), the protective and antioxidant β-cell defense must respond by first up-regulating MT and SOD, and secondarily CAT, GSH, and GPx at the correct levels by the stress or damage stimuli [106].

### 9.1. Nonenzymatic Antioxidant Defense in β-Cell

In mouse and human Langerhans islets, MT expression (constitutive and/or inducible) against oxidative stress and heavy metals has been studied for decades [107]. β-cells strongly expressed Mt1 and Mt2. The mRNA level of Mt1 is relatively higher than Mt2. In human β-cells, MT1E, MT1F, MT1X, and MT2A participate in differentiation, maturation, and other significant biological roles [107,108,109]. The MTs are the first line of defense in β-cell, when their thiols are oxidized, releasing Zn. Cytoplasmic free Zn increase activates MTF1, binding to metal response elements (MREs) and ensuing upregulation of MT gene expression [110]. MT gene expression in β-cells is also regulated by glucose [108,111,112,113,114]. Langerhans islets exposure to H_2_O_2_, IL-1β, TNF-α, and IFNγ markedly increases *Mt1a* mRNA levels [109,115]. Interestingly, Mt1a upregulation parallels oxidative stress-responsive genes, such as Cat, Gst, and Sod2 [116,117]. Evidence suggests that MT can transfer Zn to SOD1 [118]. In addition to MT in β-cells, GSH has been proposed as the primary cell defense; however, the antioxidant activity of MT is about 50 times higher against oxidative DNA damage and about 10 times higher against lipid peroxidation [119].

GSH can react directly or with enzymatic antioxidants against heavy metals, ROS, RNS, lipoperoxides, and oxidized DNA [120,121]. GSH is present in an adequate concentration to protect and detoxify β-cells. GSH and GPx activity avoids mitochondria damage in Cd presence or high glucose fluxes [122]. GST and GR activity are indispensable to maintaining GSH levels over oxidized form (GSSG). Hyperglycemia and hyperlipidemia aggravate oxidative stress in β-cells by reducing antioxidant capability, evidenced in reduced oxidized glutathione (GSH/GSSG ratio) [123]. In addition, GSH synergically acts with thioredoxins, peroxiredoxins, and other thiol-peroxidases to protect membranes and DNA from lipid peroxidation, scavenging free radicals, and xenobiotics.

### 9.2. Antioxidant Enzymes

The harmful effects of oxidative stress on β-cell death and dysfunction have been extensively discussed. β-cells have been shown to express low enzymatic concentrations of CAT, SOD, and GPx compared to other tissues such as the liver, brain, kidney, and heart. Regarding the liver, β-cells are only equipped with about 50% of the SOD and 5% of H_2_O_2_-scavenging enzymes GPx and CAT [124]. CuZn-SOD and Mn-SOD gene expression levels are 30–40% of those in the liver, whereas GPx gene expression is 15%, and CAT gene expression is barely 5% in β-cells [125]. Therefore, β-cells are highly susceptible to oxidative stress and cytotoxicity. Cadmium exposure, hyperglycemia, and hyperlipidemia (mainly FFA) provoke an overexpression of the GPx4 isoform as the stress response. A mechanism that contributes to lipid-induced oxidative stress in β-cells is the modulation of the respiratory chain by Cd (complex III), glucose, and FFA (both on complex I) in mitochondria, which seem to be the radical source [126,127,128]. Hyperlipidemia and an over-FFA uptake significantly increase superoxide anion levels and reduce antioxidative defense, suggesting that hyperlipidemia may be a driving force for ROS production. This idea is strengthened because, in the presence of high glucose levels, SOD isoforms and CAT maintain their activity. However, exposure to high concentrations of long-chain FFA results in toxic levels of H_2_O_2_ and the inability to protect β-cells [129]. Nevertheless, environmental conditions of Cd exposure have not been well-studied.

**Figure 2:**
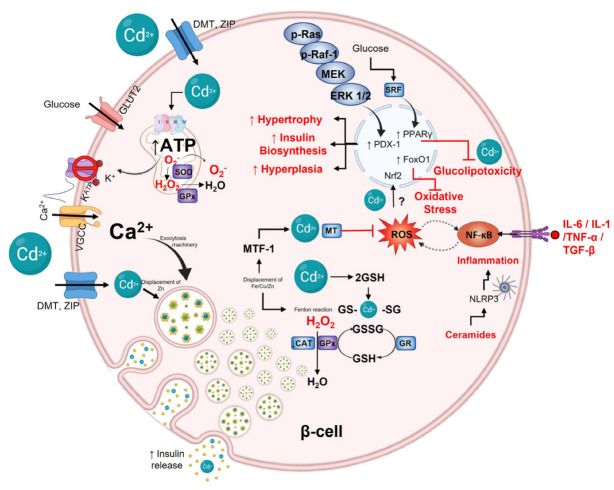
Cadmium effect on pancreatic β-cell. Glucose is the primary stimulus for insulin release. Glucose enters β-cells through GLUT2; after oxidative metabolism in mitochondria, ATP increases and then closes K^+^_ATP_ channels, depolarizing the plasmatic membrane and, consequently, opening VGCC channels, which causes calcium influx entry to the cytoplasm and activating exocytotic machinery for mobilizing and fusion insulin granules. Cadmium can make entry into the cells via DMT and ZIP transporters. Inside, Cd^2+^ can displace Zn^2+^ on insulin hexamers affecting the hormone stability and action. p-Ras, p-Raf−1m MEK, and ERK 1/2 pathway can be activated by Cd^2+^ modulating PDX−1 expression to increase insulin biosynthesis, hyperplasia, and hypertrophy to maintain hyperinsulinemia. Furthermore, glucose through SRF can activate PPARγ to protect against Cd^2+^-induced glucolipotoxicity. Cd^2+^ can interfere with redox balance in cells, generating ROS via Fenton reactions or interfering with mitochondrial complex III. When Cd^2+^ displaces Zn^2+^, it activates MTF, increasing MT and GSH expression. MT and GSH can form complexes with Cd^2+^ by sulfhydryl groups of cysteine. Finally, cytokines such as IL−6, IL−1, TNF-α, and TGF−β, as well as ceramides by NLRP3 inflammasome, activate NF-kB, which regulates and is regulated by ROS.

Redox balance is regulated at the transcriptional level in response to the harmful effects of excess nutrients or xenobiotics in β-cell. The Nrf2/Keap1 pathway regulates the expression of over 100 genes and functions related to oxidative stress and cell survival, including the enzymatic and nonenzymatic antioxidants, growth factors, and transcription factors related to inflammation [39,130]. The role of the Nrf2/Keap1 pathway in β-cells has been extensively explored in metabolic and diabetic pathologies. Nrf2 suppression increases intracellular ROS and aggravates damage in Langerhans islets and β-cell lines [131,132]. Conversely, overexpression of Nrf2 reduces DNA adduct formation and reestablishes insulin secretion [132]. However, the β-cell Nrf2 pathway has not been studied in environmental conditions of Cd exposure (Figure 2).

## 10. Cadmium and Liver

In acute Cd exposure, the liver takes up and stores a significant quantity of metal. Cd-hepatotoxicity during the initial hours after exposure produces ischemia, Kupffer cell activation, and neutrophil infiltration [133]. Liver histopathological hallmarks after acute Cd exposure are cytoplasmic vacuolization, necrosis, congested blood vessels, mitophagy, fatty droplets, glycogen depletion, and portal fibrosis [134]. However, oral subacute Cd exposure to LOAEL dose causes sinusoid obstruction of the central vein, leukocyte infiltration in portal zones, and hepatocyte hyperplasia but mild apoptotic signs [135]. Cadmium hepatotoxicity is closely related to inflammation. Previous studies have demonstrated that Cd-activated Kupffer cells release a number of inflammatory mediators, such as TNF-α, IL-1β, IL-6, and IL-8, which initiate a cascade of cellular responses, leading to liver damage [133,135]. Hepatotoxicity is reduced when Kupffer cells are selectively inhibited or suppressed [134,136]. At the same time, inflammation is accompanied by ROS, and vice versa, since ROS induces inflammation. Both cytokine and ROS signaling activate transcription factors, such as NF-κB and AP-1, which enhance the expression of pro-inflammatory and adhesion molecule genes [33,137,138].

Oxidative stress and inflammation disrupt the insulin metabolic pathway in the liver. Inflammation per se can induce insulin resistance via the MAPK pathway. Low-level Cd exposure indirectly stimulates MAPK in mice and cell cultures through oxidative stress [27,33,87]. Mechanisms include MT depletion and GSH reduction [69,139], ROS overproduction through NADPH oxidase activity [34,140], and complex III mitochondrial by accumulating unstable semiubiquinones. Increased superoxide radicals [141,142] and free iron, zinc, and selenium replacement with lost enzymatic activity (e.g., CAT, SOD, and GPx) have also been found [143,144]. Notably, oxidative stress, TNF-α, and IL-1β activate the c-Jun NH2-terminal kinase (JNK), generating insulin signaling interruption by modifying the phosphorylation of insulin receptor and insulin receptor substrate-1, provoking insulin resistance [145,146,147]. Changes in mitogen signaling consequently alter the metabolic pathway. This redundancy causes loss of glucose homeostasis and a high lipogenic rate observed in Cd-exposed animal models.

## 11. Cadmium Impairs Hepatic Glucose Homeostasis

Studies investigating the impact of environmentally relevant doses of Cd showed sex specificity in glucose metabolism disruption, with females being more sensitive [148,149]. Low-level maternal Cd exposure influences glucose homeostasis in offspring and increases the risk of developing T2D [128]. Chapatwala et al. demonstrated that Cd administration in environmental and toxic dosages for 30 days increased pyruvate carboxylase, phosphoenol pyruvate carboxykinase, fructose-1, 6-diphosphatase, and glucose-6-phosphatase in the liver, which indicates that Cd may induce gluconeogenesis from noncarbohydrate sources [148,149]. Additionally, Cd can alter PI3K/Akt/mTOR signaling, interfering in metabolic actions such as glycolysis and glycogenesis, promoting gluconeogenesis and glycogenolysis via dysregulation of FOXO1 and glycogen synthase kinase-3β (GSK3β) [150,151,152,153]. Hepatic glycogen synthesis results from postprandial insulin signaling [154,155]. Phosphorylation-based glycogen synthase (GS) regulation by insulin occurs through Akt phosphorylation and GSK3β. Thus inactivated, GSK3β kinase activity toward GS is diminished, and dephosphorylated GS is, in turn, more active. In hyperglycemic states associated with insulin resistance, GSK3β is overactivated, which impedes glycogen synthesis. Likewise, Cd exposure seems to be an inductor of the activity of GSK3β or an inhibitor of GS because animals exposed to a chronic environmental LOAEL dose show insulin resistance and continuous reduction in hepatic glycogen levels [77,135,156,157].

## 12. Cadmium, De Novo Lipogenesis, and Dyslipidemia

De novo lipogenesis (DNL) is a natural pathway to control glucose levels and energy storage, encouraging hepatic triglyceride biosynthesis, which is dependent on proper insulin signaling. This process is an extension of the complex metabolic networks and is provided with substrate primarily through glycolysis and the metabolism of carbohydrates. However, when carbohydrate pathways are impaired, hypertriglyceridemia and breaking the balance between lipogenesis and lipolysis have been observed as common factors in physiopathological processes such as insulin resistance, obesity, and T2D [158]. Regulation of the lipogenic pathway is in two levels, first by transcriptional regulation of enzymes integral to fatty acids (FA) synthesis, and second by allosteric regulation of acetyl-CoA carboxylase (ACC). The transcriptional regulation of DNL by sterol regulatory element-binding protein 1c (SREBP1c) is activated via PI3K/Akt/mTOR (insulin signaling), whereas increased glucose concentrations activate the carbohydrate response element-binding protein (ChREBP); both are induced by feeding [158,159,160]. The acetyl-CoA produced during glycolysis through ACC activity generates malonyl-CoA that is incorporated into the synthesis FA and then, through the initial acylation of glycerol-3-phosphate, rendered in consecutive cycles, mono, di, and triacylglycerol. Triglycerides interact with microsomal triglyceride transfer protein (MTP), which aids the partial lipidation of the nascent apolipoprotein B 100, a hallmark of very low-density lipoproteins (VLDL). MTP and apoB100 are modulated via FoxO1 through insulin signaling. Therefore, both hyperinsulinemia and insulin resistance overfeed the lipogenic conditions. It is named “the lipid paradox,” characterized by hypertriglyceridemia in fasting and postprandial states [158].

The oral cadmium LOAEL dose exposure results in a GSK3β decrease, providing excess lipogenic substrates. In addition, SREBP-1c is strongly expressed in the liver of environmental Cd-exposure animal models [135]. The evidence suggests that hepatic Cd accumulation induces insulin resistance, favors DNL, and modifies the lipidome serum and hepatic system [76,77,157]. The lipidome is the content, distribution, and type of lipids in tissue or lipoproteins. The overproduction of triglyceride-rich lipoproteins (TRLs) would be a straightforward consequence of the metabolic toxicity caused by Cd in the liver. In particular, rats exposed to Cd LOAEL dose overproduce large VLDL (VLDL1) [76]. VLDL1 is a TRL, and its formation is highly dependent on hepatic triglyceride accumulation (cytosolic lipid droplets) and FFA fluxes from serum to the liver [161,162]. Previous works have shown that chronic cadmium LOAEL dose exposure develops hepatic steatosis and small low-density lipoproteins (sLDL) [76]. VLDL1 presence is a prerequisite for forming sLDL [163,164]. The combination of sLDL and insulin resistance is a factor risk to atherosclerosis development [76,160]. Experimental evidence suggests that Cd could contribute to the initiation of atherosclerosis and promote the progression of cardiovascular disease through damage, stress, and inflammation. In addition, environmental Cd exposure generates small HDL sub-fractions (HDL-3c) [76]. These sub-fractions present low content of free and esterified cholesterol and phospholipids but are rich in triglycerides. Together, hypertriglyceridemia, low cholesterol level in HDL, and augmented FFA concentration are a consensus of metabolic dyslipidemia. Therefore, Cd exposure can be a risk factor for multi-tissular steatosis, atherosclerosis, and metabolic dyslipidemia associated with defects in the enzymatic activity of cholesterol ester transfer protein, lecithin-cholesterol acyltransferase, and phospholipid transfer protein (PLTP) [165]. Nevertheless, only a lack of PLTP activity has been observed after Cd administration [166]. Likewise, triglyceride-rich HDL3c could be a promoter of oxidative stress and inflammation in the liver and other tissues [167].

## 13. Hepatic Antioxidant Defense

The liver has a robust defense system to prevent injury associated with Cd exposure or other attacking molecules. The studies of Cd exposure in experimental animals and in vitro cellular models have yielded substantial information regarding metal toxicity and cellular defense mechanisms. Concerning mechanisms postulated to prevent hepatotoxicity, the literature mentioned the activity of sulfhydryl group binding, implicating membrane proteins, cytoplasmic proteins, and enzymes [168]. In addition, the interaction with an excessive quantity of essential metals such as zinc, selenium, copper, and calcium maintains low Cd toxicity [169]. Metal homeostasis is critical for many enzymes, transcription factors, and other subcellular proteins [75]. The substitution of native metals by Cd is often proposed as the leading molecular mechanism of Cd hepatotoxicity. In hepatocytes, Cd uptake and accumulation are mediated by transporters on essential metals [169]. Calcium channels and carriers actively participate in Cd uptake because they interact with sulfhydryl groups [170,171]. Furthermore, membrane transporters such as DMT1, ZIP8, and ZIP14 play a role in Cd uptake [11,172]. It has been shown that Cd-ferritin and Cd-MT complexes may enter hepatocytes by receptor-mediated endocytosis [173]. Numerous experiments have demonstrated that the isoform presence of MT-1/-2 shields cells from Cd toxicity [69,171,174,175].

Metallothionein and GSH are primary line defenses that maintain the hepatocytes’ redox state and, consequently, prevent inflammation, xenobiotic injury, metal toxicity, and many harmful effects. Complex formation between Cd and GSH has been implicated as the initial step in hepatic detoxification before transferring the metal to MT and other cysteine-rich peptides [176]. In chronic Cd exposure, the redox status is compromised. In cells and primary rat hepatocyte cultures, Cd-induced GSH loss and cell death indicate that GSH is critical in cell function and survival [142]. GSH administration not only reduces the generation of free radicals but also lowers the accumulation of Cd in the liver [177]. Hepatic Cd accumulation, metabolic impairment, dyslipidemia, and steatosis caused by metal establish an oxidative status and ROS generation in hepatocytes that activate hepatic stellate cells and Kupffer cells that favor a profibrotic status. Therefore, various cellular defense mechanisms could be started. Enzymes and other antioxidant components constitute the system to defend against Cd-induced oxidative damage [38]. In normal conditions, GSSG is regenerated to GSH by the action of GR; however, Cd inhibits GR activity, which induces mortality and hepatotoxicity. Furthermore, the electrophilic sites of oxidized molecules are neutralized by GST and the thiol group of GSH, thereby rendering the products more water-soluble and less toxic. It has been reported that Cd decreases hepatic GST activity [178,179].

Although the liver is one tissue that expresses a high concentration and activity of detoxifying and antioxidant enzymes, Cd accumulation induces high oxidative stress and lipid peroxidation levels, thereby gradually decreasing their activity [180,181,182]. The most affected enzymatic activity observed after chronic environmental Cd exposure occurs in SOD, CAT, and GPx [183]. It has been proposed that the inactivation of the SOD isoforms results from a displacement of Cd by Zn or Mg from the catalytic domine through competitive inhibition or substitution [184]. However, the results depend on Cd concentration and exposure time.

An acute administration of 5 mg/kg decreases SOD activity [185]. In contrast, by 12 weeks, oral Cd exposure (50 ppm) increases hepatic SOD activity [51]. Likewise, the inhibition or reduction in GPx activity has been associated with Se depletion through Cd–Se–cys complex formation at the enzyme’s active site [186]. It has been suggested that competition between GPx and MTs for S-amino acids is a potential cause of GPx activity decrease during Cd stress [54,178]. Additionally, CAT activity increases after six days of daily intraperitoneal Cd administration or five days via gastric gavage in environmental concentrations but decreases its activity during acute intraperitoneal administration in toxicological levels [184,187,188]. Possible underlying mechanisms for decreased CAT activity have been postulated, such as the interaction between Cd and the catalytic subunit of CAT, Fe deficiency, since CAT has Fe as an essential element in its active center, and displacement of Fe from the catalytic domine [51].

**Figure 3:**
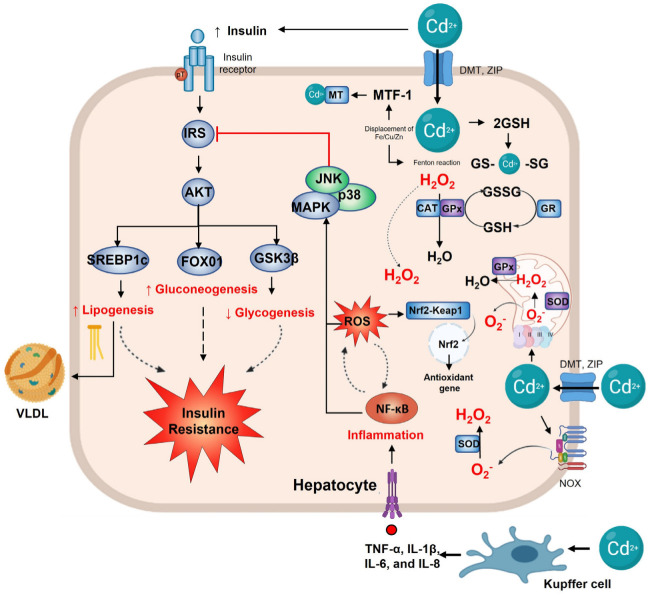
Hepatic alterations in cadmium exposure. In the hepatocyte, the main effect of the insulin pathway is to regulate metabolism; when Cd^2+^ enters the cell, it disrupts the insulin signaling, increasing SREBP1c and FOX01 expression, thus, lipogenesis and gluconeogenesis. At the same time, it decreases GSK3β and glycogenesis, which leads to insulin resistance. Cadmium exposure activates the Kupffer cell, increasing TNF−α, IL-1γ/β, IL−6, and IL−8. Upregulated inflammation and NF−κB activate p38 and the JNK pathway to inhibit IRS and insulin signaling. Cd^2+^ also induces oxidative stress by the generation of •O_2_^-^ via NOX or mitochondrial complex III. Mn−SOD and Cu/Zn-SOD play a vital role in detoxifying it, forming H_2_O_2_ that can be converted to H_2_O by GPx or CAT. The increased ROS by Cd^2+^ induces the Nrf2 gene to activate antioxidant gene transcription. On the other hand, Cd^2+^ can displace Fe, Cu, and Zn, leading to the Fenton reaction and producing more ROS. Zn^2+^ displacement activates MTF−1, which leads to GSH formation for Cd^2+^ detoxification.

Enzymatic and nonenzymatic antioxidant systems are essential to hepatic function in physiological or pathological conditions [189,190]. The hepatocytes sense redox balance via Nrf2. The Nrf2 activity increases antioxidant systems in different organelles. Notably, in mitochondria, Nrf2 activity protects against permeability, maintains the redox equilibrium, and enhances mitochondrial biogenesis by promoting the transcription of nuclear respiratory factor 1 (Nrf1). Therefore, Nrf2 protects mitochondria from oxidative stress. Correct mitochondrial function avoids hepatic steatosis, facilitating fatty acid metabolism by directly regulating related genes [190,191]. In hepatocytes, Cd stabilizes Nrf2, prevents degradation, and promotes nuclear translocation as a protective mechanism [192]. Cd exposure modulates NQO1 and HO-1 hepatic expression via Nrf2 [193,194]. Additionally, it has been speculated that through a specific G protein-coupled metal-binding receptor, Cd induces phospholipase C activity, releases intracellular Ca^2+^ and diacylglycerol, and provokes nuclear Nrf2 activation [195]. Furthermore, Zn displacement from MT by Cd induces the Nrf2 signaling pathway, which has two effects: suppresses inflammatory responses and enhances the antioxidant defense system by MTs upregulation. Consequently, it protects Keap1–Nrf2 oxidization upon Cd exposure [196,197]. Acute administration of a single CdCl_2_ dose in toxic concentration inhibits Nrf2 and HO-1 and activates inflammatory signaling pathways, NF-κB, NLRP3, and MAPK [194]. The absence or inhibition of Nrf2 causes a dramatic ROS elevation and, consequently, cell death [198,199] (Figure 3).

## 14. Cadmium and Adipose Tissue

Adipose tissue is the main body energy reservoir and can store Cd [200]. In addition, adipose possesses endocrine functions, secreting adipokines, growth factors, cytokines, and chemokines [201]. Adipokines are mediators of various metabolic processes such as fatty acid oxidation, DNL, gluconeogenesis, glucose uptake, insulin signaling, and energy expenditure in metabolically active tissues such as the liver, skeletal muscle, and brain [201,202,203]. Clinically, adiponectin and leptin are the most important adipokines [204]. The endocrine function of adipose tissue is regulated significantly by the nutritional status linked to energy storage [205]. White adipocytes are tasked with storing excess energy as triglycerides. These undergo hyperplasia to increase the number of adipocytes and hypertrophy to increase the size of each adipocyte, allowing fatty tissue to expand when there are excessive nutrients [206]. As needed, during fasting and exercise, triglycerides stored in adipocytes are mobilized to provide fatty acids for energy utilization by the rest of the body. Stored triglycerides are in a constant state of flux, whereby energy storage and mobilization are determined mainly by the energetic necessity and hormonal fluctuations [207]. Therefore, through adipokines, adipose tissue cross-talks and regulates other body organs, metabolism, immunity, reproduction, and cardiovascular functions [206,208].

In white adipose tissue (WAT), Cd accumulation is also dose- and time-dependent [135,156]. Different concentrations of subcutaneous Cd administrations (3.5 times/week) for two weeks showed Cd accumulation in epididymal adipose tissue and abnormal adipocyte differentiation, expansion, and function [209,210]. In toxicological doses, adipocyte size is reduced, even in chronic exposure [211]. However, in chronic environmental exposure conditions, Cd was accumulated in retroperitoneal adipose, and histological analysis revealed cellular hypertrophy with a reduced adipocyte number per field. The hypertrophy was associated with increased triglyceride concentration in adipose tissue, fasting, and after insulin administration; however, a sustained lipolysis rate was observed, as was evidenced by serum FFA concentration [135]. The toxicological studies showed that WAT exposed to Cd reduces the expression level of mRNA and protein of peroxisome proliferator-activated receptor-gamma (PPAR-γ) and the protein of CCAAT/enhancer-binding protein (C/EBP), which suggests an adipogenesis impairment, but functional capacity and plasticity features [209,210,212]. Hypertrophy is a plasticity feature of adipocytes to store triglyceride. It is also an obesity-independent marker of insulin resistance and indicates a future risk of developing metabolic diseases driven by limited hyperplastic expansion and storage capacity [213,214]. Adipose tissue insulin resistance is an early hallmark of metabolic alteration, evidenced in chronic and subacute Cd exposure. A tyrosine phosphorylation reduction accompanies adipose insulin resistance on the insulin receptor [77,135]. Reduced signaling in the adipocyte metabolic pathway caused by Cd exposure has multiple consequences. For instance, insulin-stimulated glucose uptake in adipocytes depends on PI3K-Akt proper signaling [215,216], supporting GLUT4 trafficking to the plasma membrane [217], and glycogen synthesis. Additionally, the correct activation of PI3K-Akt has antiapoptotic and anti-inflammatory effects [218,219]. Chronic Cd exposure has been associated with diminished insulin receptors in rat adipocytes, which is related to the diabetogenic effect [220].

Adipocyte hypertrophy inevitably reaches a limit at which additional anabolic pressure is excessive due to cell expansion limitations. Adipocytes reach this threshold and initiate an inflammatory response [221]. Pro-inflammatory cytokines, such as TNF-α, inhibit adipocyte differentiation, increase lipolysis, interfere with insulin signaling, and induce a macrophage-like phenotype in preadipocytes [214,222]. TNF-α also induces increased IL-1β and IL-6, which polarizes adipose tissue macrophages, increasing subclass M1 recruitment that releases and produces more TNF-α, IL-6, IL-12, IL-23, and IL-1β. Meanwhile, M2 macrophages diminish, as well as IL-10, IL-4, and TGF-β cytokine levels [223,224]. Several findings showed that Cd activates the NF-κB pathway in adipocytes, leading to the upregulation of pro-inflammatory cytokines [20,225]. Studies have also demonstrated reliable data identifying chronic low-grade inflammation as an essential link between systemic insulin resistance and metabolic diseases. These effects include the activation of NF-κB and inhibition of AMPK signaling, leading to impaired insulin homeostasis [226]. Serum concentrations of several pro-inflammatory cytokines inhibit insulin signaling, which results in signal redundancy since insulin resistance induces inflammation and vice versa [227]. In addition, Cd exposure and inflammation negatively affect adipokine secretion. Subcutaneous Cd administrations have shown adipokine diminishing dose dependently [209,210]. However, chronic environmental exposure conditions increase leptin release, even associated with insulin concentration, while serum adiponectin concentration did not show changes [135]. Adiponectin plays an anti-inflammatory role and has insulin-sensitizing effects [228,229]. Moreover, adiponectin is a biomarker of healthy adipocyte expansion and prevents steatosis risk [230]. Meanwhile, leptin regulates food intake, lipid metabolism, energy expenditure, and glucose homeostasis [231]. Leptin releasing is proportional to adipocyte size; adipocyte hypertrophy carries hyperleptinemia, whereas lipodystrophy observes hypoleptinemia. The disturbance of insulin, adiponectin, and leptin contribute to metabolic diseases [135]. However, how or what Cd does in adipocytes impairment adipokine production and release have not been fully understood.

## 15. Adipose Tissue Antioxidant Defense

It has been shown that obesity contributes to the development of systemic oxidative stress in both human and animal studies [232,233]. Oxidative stress markers, such as protein carbonyls, lipid peroxidation products, and ROS production, increase in adipose tissues of obese subjects [234]. In the db/db mice model, ROS production and lipid peroxidation accumulation were also upregulated in adipocytes [235]. Possible triggers of obesity-induced oxidative stress include altered nutritional status, hyperglycemia, insulin resistance, hyperlipidemia, and low-grade chronic inflammation [236]. TNF-α, IL-6, and IL-1β signaling increase ROS production in the adipose tissue of obese individuals [37,237]. In cell cultures, 3T3-L1 adipocytes treated with TNF-α decreased the expression of antioxidants, such as GST, peroxiredoxin, and GPx, resulting in protein carbonylation, ROS generation, and mitochondrial dysfunction [238,239]. In adipocytes, ROS can be produced by NADPH oxidase (Nox), xanthine oxidases, and mitochondria. In particular, isoform Nox4 activity has been found in obese mice [240]. ROS production is induced by glucose and palmitate via Nox4 rather than mitochondrial oxidation. Likewise, xanthine oxidase increases activity in obese subjects, generating H_2_O_2_ that potentially links adipocyte DNL with ROS production [237]. Oxidative stress in adipose tissue activates NF-κB and MAPK, downregulating anti-inflammatory adipokines and adiponectin production while upregulating pro-inflammatory cytokines [232,240,241].

**Figure 4:**
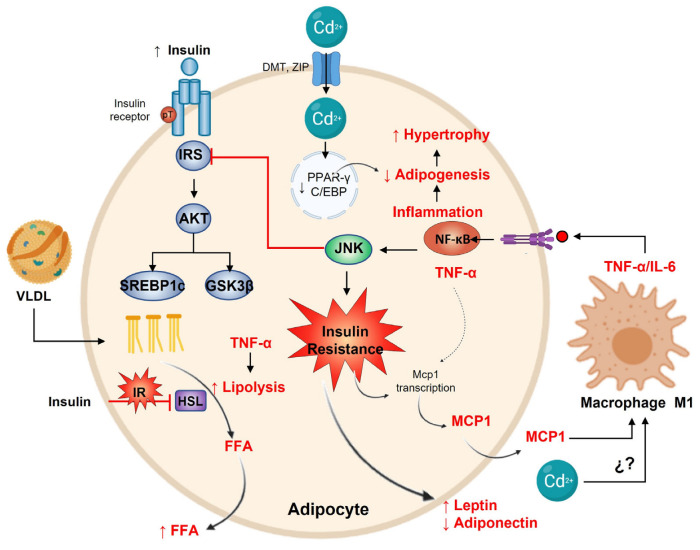
Adipocyte response to cadmium. The prime function of adipocytes is to store lipids in response to SREBP1c with the insulin pathway. Contrarily, Cd^2+^ decreases adipogenesis by reducing PPARγ and C/EBP expression. Insulin resistance promotes MCP-1 and macrophage M1 differentiation with the increase in TNF-α and IL-6 secretion, leading to the adipocyte inflamed. The inflammation activates NF-kB, which in turn regulates adipogenesis and hypertrophy. On the other hand, TNF-α promotes lipolysis with FFA liberation (the original opposite effect of insulin signaling) and MCP-1 transcription. Finally, on insulin resistance, adipocytes increase leptin and decrease adiponectin release.

The antioxidant defense in white adipose tissue is limited; thereby, ROS accumulation contributes directly to the metabolic imbalance (impairs glucose uptake, over-release of free fatty acids, and jeopardizes mitochondrial biogenesis and functions) [242,243], linking excessive nutrient stress, Cd exposure, and insulin resistance [244,245]. Studies have shown that ROS production increases parallel with adipocyte fat accumulation; consequently, adipose tissue is a significant source of plasma ROS [246]. In the adipocytes, the activity and expression of SOD1, CAT, peroxiredoxins, and GPx have been reported. However, the antioxidant defense of adipose tissue concerning Cd exposure has yet to be well-studied. It has been found that toxic concentration of Cd exposure by oral gavage showed an activity decrease in SOD, CAT, and GPx [247]. MTs play a critical role in adipogenesis and maintaining adipocyte size beyond their chelating capacity [210]. On the other hand, in the adipocyte insulin resistance of obese mice and humans, antioxidant peroxiredoxin 3 (Prdx3) decreases significantly. Mitochondria are severely affected when Prx3 falls because H_2_O_2_ induces mitophagy [248]. Obesity-induced inflammation also decreases the expression of Prdx3 and GPx in epididymal adipose tissue [244]. Prdx3-deficient adipocytes exhibit high superoxide production, decreased mitochondrial potential, and altered adipokine expression. Prdx3 knockout mice observed adipocyte hypertrophy and fat overstorage [248]. Meanwhile, the Prdx2 isoform seems to participate in adipocyte differentiation because its silencing inhibits adipogenesis and increases ROS production in the 3T3-L1 cell line [249]. Obese models that augment ROS production present a low rate of GSH synthesis and reduce CAT and SOD1 activity and insulin sensibility [250]. Likewise, adipocyte-specific HO-1 knockout causes hyperglycemia and hyperinsulinemia in female mice but not male mice, indicating a sex-depended protective role [251]. The adipocyte-specific overexpression of HO-1 attenuated high-fat diet-induced adiposity and inflammation, improving insulin sensitivity and adiponectin levels [252] (Figure 4).

## 16. Cadmium and Obesity

Based on the relationship between heavy metals such as lead and obesity [253,254], the role of cadmium in developing this pathology has also been studied [255]. Despite multiple studies showing an association between environmental exposure to Cd and obesity, data are inconsistent. Cadmium exposure during the prenatal period has long been associated with lower birth weight and gestational age [256,257,258]. Low birth weight, often followed by rapid adiposity gain, is a consistent risk factor for cardiovascular and metabolic impairment later in life [259]. In the juvenile stage, an association exists between higher body weight, waist, and hip circumference in young females (8–15 years old) with high blood Cd levels [260]. In adulthood, Haswell-Elkins et al., 2007, showed a positive correlation between Cd levels and waist circumference but not with body weight [261]. Skalnaya et al., 2014, reported in women aged 22–35 years a weak but significant correlation between hair Cd concentration and high body weight values [262], whereas Filippini et al., 2016, demonstrated this association in Italian females but not in men [263]. The report on associations of cumulative exposure to heavy metal mixtures with obesity and its comorbidities among US adults in NHANES 2003–2014 showed a correlation between Cd serum and urinary concentration with higher body mass index, concluding that cumulative exposure to heavy metals as mixtures is associated with obesity and its related chronic conditions such as hypertension and T2D [264]. A study that included 5544 Chinese adults evaluated blood Cd levels related to diabetes or obesity. Results showed a positive relationship between blood Cd levels, fasting hyperglycemia, and prediabetes prevalence, but not with obesity [265].

In support of these human observations, animal studies have shown that early-life Cd exposure also increases fat mass, adiposity, and body weight in male mice [266,267]. Similar results have been found in chronic Cd exposure at LOAEL dose for five months. Male Wistar rats show a higher body weight, abdominal perimeter, BMI, and fat percentage, when exposed to an LOAEL dose from early-life stage [156]. However, young adult female and male rats exposed to a subacute and acute oral environmental amount of Cd resulted in low body weight, the delta of weight gain, and abdominal perimeter [131,268]. Regardless of the time, route of administration, concentration, or gender of experimental Cd exposed animals, adipose tissue shows significantly impaired physiology and function.

## 17. Cadmium and Diabetes

The evidence indicates that Cd exposure by direct and indirect mechanisms disrupts the pancreas–liver–adipose axis and produces a metabolic impairment, clinically exhibiting dysglycemia, hyperinsulinemia, insulin resistance, and dyslipidemia. These parameters are associated with metabolic syndrome development and its complications, such as atherosclerosis, hypertension, cerebrovascular and cardiovascular diseases, β-cell exhaustion, nonalcoholic fatty liver and its progression to fibrosis or cirrhosis, and prediabetes and diabetes. Therefore, Cd accumulation shows a diabetogenic effect in short-term acute exposure and long-term chronic expositions [77,269,270]. The mechanism(s) by which Cd may alter glucose homeostasis involves changes in glucose transporter expression, gluconeogenesis, and metabolism mediators impairment (leptin, glucose-dependent insulinotropic polypeptide, and pancreas polypeptide) [148,219]. However, impaired insulin levels after glucose stimulation and pancreatic β-cell dysfunction are likely Cd toxicity factors. In vitro and in vivo, Cd accumulates within human pancreatic β-cells and alters glucose-stimulated insulin release [269,271,272].

Epidemiological studies have also shown that subjects with occupational exposure to Cd have signs of prediabetes [273,274]. A positive association between Cd concentrations in urine (2–4.0 µg/g creatinine) and blood (1.2–2.5 µg/L) with prediabetes or diabetes [275,276]. Different studies suggest that risk increases linearly up to approximately two µg/g of creatinine. Swaddiwudhipong et al., 2012, found that persons occupationally or environmentally exposed to high Cd levels in Northwestern Thailand had a significant prevalence of diabetes [277]. Likewise, Zhang et al. 2014, showed hyperglycemia in residents in a polluted area in China [278]. Another study by Tangvarasittichai et al., 2015, demonstrated that Cd exposure was associated with a higher prevalence of diabetes [279]. In the prospective study realized by Xun et al., Cd exposure was related to the development of diabetes [280]. Using National Health and Nutrition Examination Survey (NHANES) data, urine Cd was positively associated with diabetes by Wallia et al. [281]. In the same way, the most compelling evidence came from a cross-sectional study by Menke et al. (2016). They used data from NHANES (1999–2010) to assess whether increased urinary Cd was associated with impaired T2D in the United States [282]. Despite evidence about Cd and diabetes development, some studies have revealed no relationship [283,284,285].

## 18. Final Remarks

Cadmium at environmental or toxic levels is accumulated in multiple tissues, depending on the time and route of administration or exposure, dose, interaction with biometals, previous diseases or comorbidities, age, and gender of population analyses. The pancreas–liver–adipose axis is susceptible to Cd accumulation and produces clinical disruptions such as metabolic syndrome. The molecular mechanisms are not yet fully understood; however, oxidative stress and inflammation are common features. Both are described in metabolic disorders and thereby link closely with diabetes progression. Each tissue of the axis possesses a different threshold for intracellular Cd management. Of course, the liver has a robust defense; contrarily the pancreas is very susceptible to Cd effects. It can be hypothesized that both pancreas and fatty tissue in the environmental conditions of Cd exposure generate hyperplasia or hypertrophy as adaptation mechanisms, but only a few works approach this topic. Hyperplasia and hypertrophy in both tissues also cause endocrine impairment, such as hyperinsulinemia that progresses to insulin resistance and long-term β-cell exhaustion. At the same time, adipocytes observe differential secretion of leptin and adiponectin, which alters the axis’ and other tissues’ metabolic functionality. The population risk analyses must consider these clinical data to clarify the link to diabetes. Meanwhile, evidence strongly suggests that Cd should be considered a risk factor for developing metabolic diseases. Future research must focus on molecular mechanisms that affect the pancreas–liver–adipose axis to find therapeutical strategies that minimize metabolic alterations.

## Data Availability

No new data were created or analyzed in this study. Data sharing is not applicable to this article.
